# The impact of ageing reveals distinct roles for human dentate gyrus and CA3 in pattern separation and object recognition memory

**DOI:** 10.1038/s41598-017-13853-8

**Published:** 2017-10-25

**Authors:** Serena E. Dillon, Demitra Tsivos, Michael Knight, Bryony McCann, Catherine Pennington, Anna I. Shiel, Myra E. Conway, Margaret A. Newson, Risto A. Kauppinen, Elizabeth J. Coulthard

**Affiliations:** 1 Bristol Institute of Clinical Neuroscience, North Bristol NHS Trust, Southmead Hospital, Bristol, BS10 5NB UK; 2 School of Clinical Sciences, University of Bristol, Learning and Research Building, Bristol, BS10 5NB UK; 3 School of Experimental Psychology, University of Bristol, 12A Priory Road, Bristol, BS8 1TU UK; 4 Department of Applied Science, University of West of England, Frenchay Campus, Coldharbour Lane, Bristol, BS16 1QY UK

## Abstract

Both recognition of familiar objects and pattern separation, a process that orthogonalises overlapping events, are critical for effective memory. Evidence is emerging that human pattern separation requires dentate gyrus. Dentate gyrus is intimately connected to CA3 where, in animals, an autoassociative network enables recall of complete memories to underpin object/event recognition. Despite huge motivation to treat age-related human memory disorders, interaction between human CA3 and dentate subfields is difficult to investigate due to small size and proximity. We tested the hypothesis that human dentate gyrus is critical for pattern separation, whereas, CA3 underpins identical object recognition. Using 3 T MR hippocampal subfield volumetry combined with a behavioural pattern separation task, we demonstrate that dentate gyrus volume predicts accuracy and response time during behavioural pattern separation whereas CA3 predicts performance in object recognition memory. Critically, human dentate gyrus volume decreases with age whereas CA3 volume is age-independent. Further, decreased dentate gyrus volume, and no other subfield volume, mediates adverse effects of aging on memory. Thus, we demonstrate distinct roles for CA3 and dentate gyrus in human memory and uncover the variegated effects of human ageing across hippocampal regions. Accurate pinpointing of focal memory-related deficits will allow future targeted treatment for memory loss.

## Introduction

**Figure 1:**
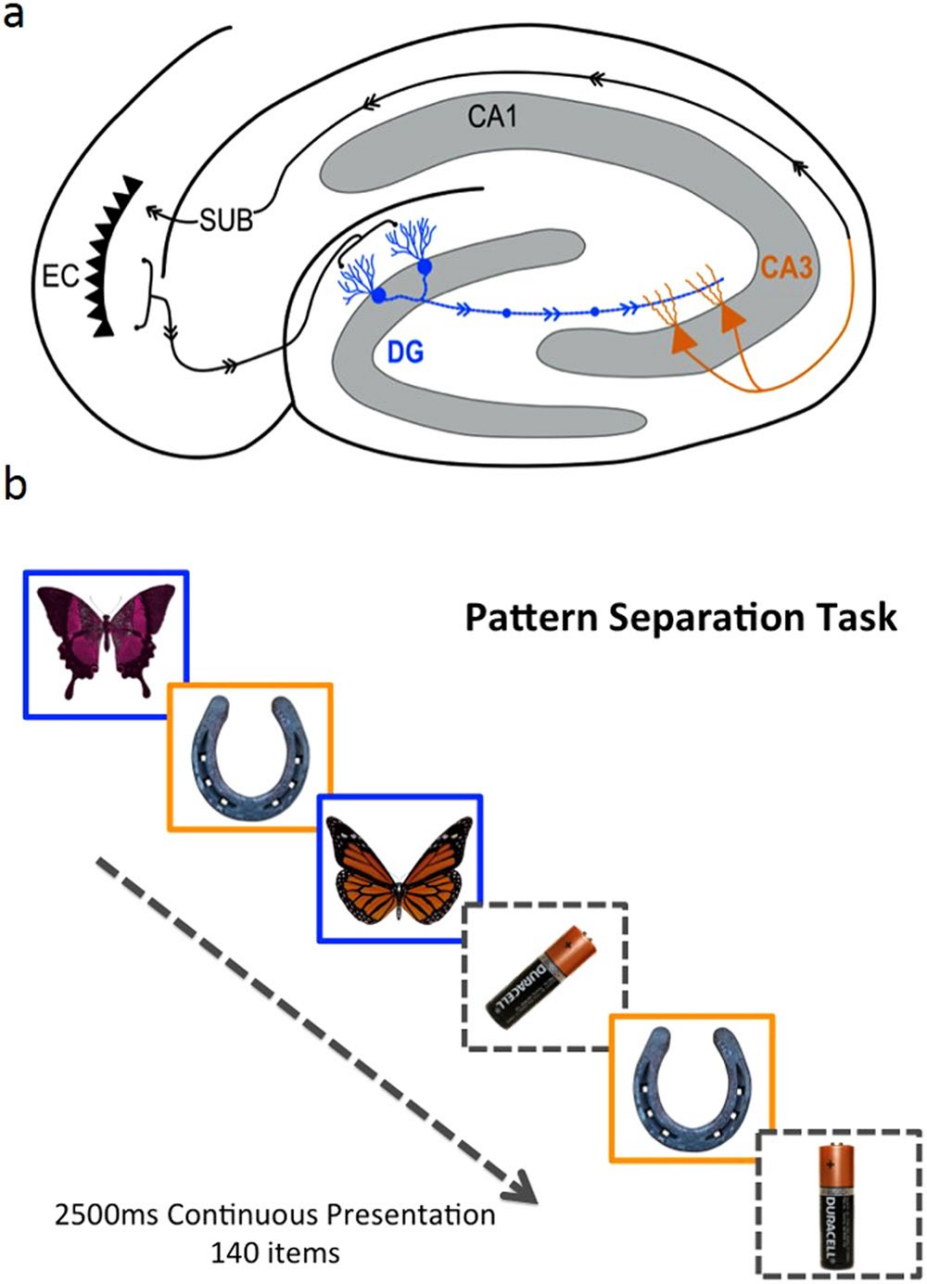
Diagram of hippocampal cross-section showing putative regions involved in pattern separation (blue) and object recognition (orange) (**a**) and behavioural task of pattern separation (blue) and object recognition (orange) (**b**). Stimuli presented are *simil*ar (blue outline in **b**) and require pattern separation thought to occur in dentate gyrus granule cells and mossy fibre network (blue in **a**), *identical* (orange outline in **b**) requiring object recognition in CA3 neurons (orange neurons in **a**), *rotated* or *new*. Throughout the paper, data are presented for similar items as a measure of pattern separation, identical items as a measure of object recognition and rotated items as a measure of detection of coordinate remapping. However, data in the new item category were too clustered around perfect performance to obtain meaningful results and are therefore not analysed. Participants respond similar, identical rotated or new to each item presented on a computer screen. DG = Dentate gyrus, SUB = subiculum, EC = Entorhinal cortex, NC = Neocortex.

Accurate memory requires both recognition of previously encountered stimuli and the ability to distinguish between similar, but distinct, events (pattern separation)^1^. In animals, recurrent collaterals in the CA3 subfield of hippocampus form an auto-associative system, permitting recall of previously stored objects and events^2^, even when only partial information is available. Dentate granule cells receive input from entorhinal cortex and outputs from dentate gyrus are more divergent than inputs^3,4^. Through such separation of overlapping inputs, the dentate gyrus is thought to underpin pattern separation^3^. Dentate granule cells form strong and sparse connections through the mossy fibre pathway projecting onto the CA3 (Fig. 1a). Thus, animal work suggests that CA3 and dentate gyrus enable accurate object recognition and pattern separation respectively. One simplified view of this network is that pattern separation in the dentate gyrus helps separate memories of similar events to protect against erroneous object recognition in CA3. However, confirmation of these roles for dentate gyrus and CA3 in humans is currently lacking.

Structural and functional magnetic resonance imaging has revealed details of hippocampal function in humans, but distinguishing between CA3 and dentate gyrus has generally been limited by spatial resolution and spatial specificity due to macrovascular contributions to BOLD fMRI^5–8^. Focal lesion studies are central to animal work as they show a brain area is *critical* for, rather than just active during, a given function, but are scarce within the human hippocampal subfield literature, as individual subfields are small^9,10^ and therefore rarely affected by disease processes or surgery in isolation.

In this study, we exploit the principle that aging is associated with differential performance on cognitive tasks – with memory biased away from pattern separation towards pattern completion^11,12^. Refinement of this theory suggests that the age related impairment of pattern separation stems from perceptual rather than conceptual difficulties^13^. In addition, there is a putative selective hippocampal subfield volume loss with ageing. In rodents and monkeys, dentate gyrus volume is proposed to be particularly reduced with advancing age^14–16^. Despite an extensive body of evidence in support of the relationship between age and loss of structural integrity in the animal dentate gyrus^15^, the relationship between human dentate gyrus and age has been controversial; however, overall, at least in later life, dentate gyrus volume appears more affected by age than CA3 volume^16–22^. Taking advantage of distinct susceptibility to age and other constitutional sources of variability of dentate gyrus and CA3 volume, we investigate the function of the dentate gyrus and CA3 across a large number of individuals.

Specifically we propose that accuracy of separation performance is defined by the volume of dentate gyrus. In contrast, we expect that pure object recognition will be CA3-dependent and CA3 volume will predict object recognition performance more precisely than dentate gyrus volume.

**Figure 2:**
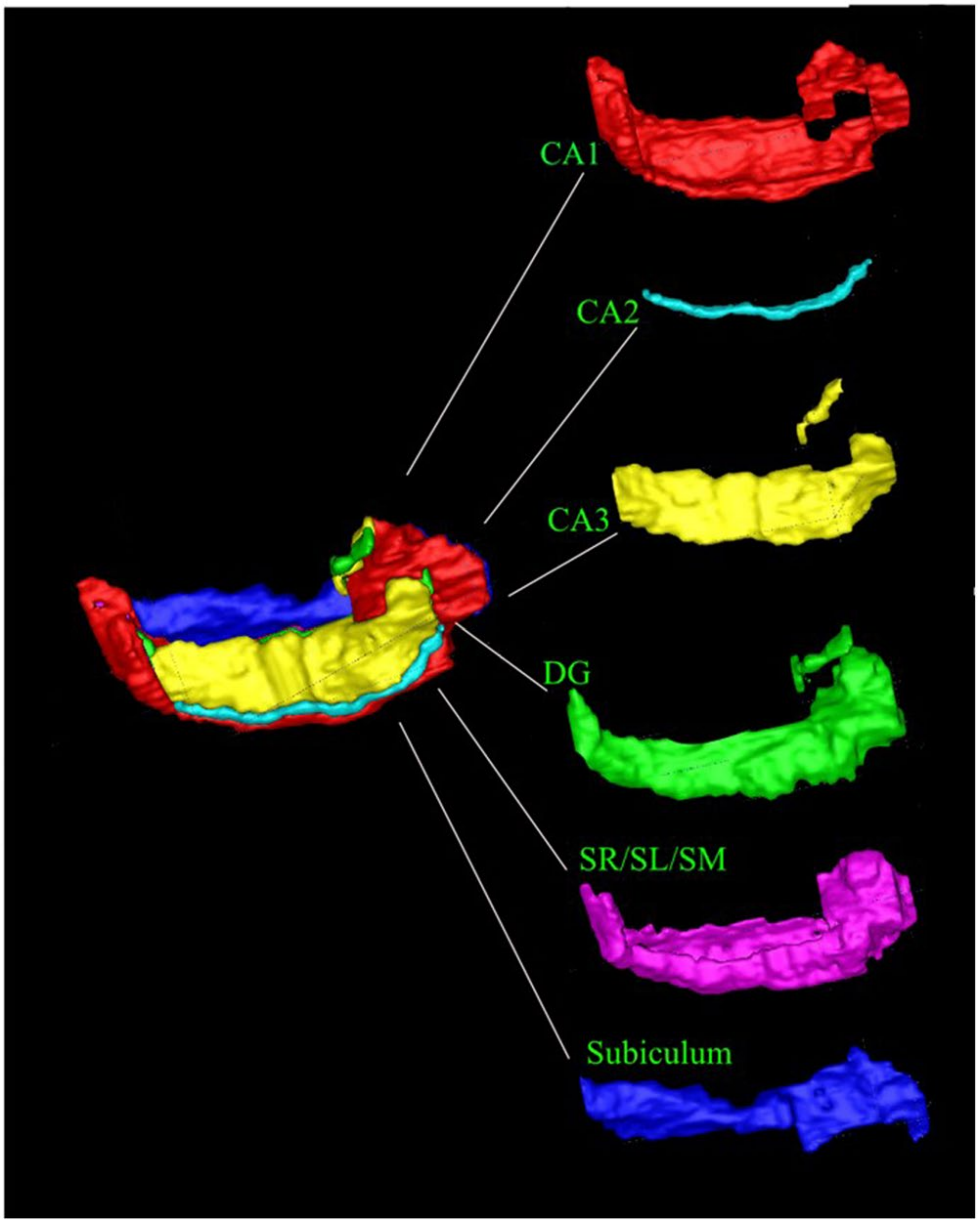
Hippocampal Segmentation. Our hippocampal segmentation protocol identifies CA1, CA2, CA3, Dentate Gyrus (DG), Stratum Radiatum/Stratum Lacunosum/Stratum Moleculare (SR/SL/SM) and subiculum. Table S1 shows subfield volumes.

We tested the neuroanatomical basis of human memory by manually segmenting hippocampal subfields (CA1, CA2, CA3, subiculum and dentate gyrus) on 3 T MRI structural scans^23^ of 65 healthy or with Mild Cognitive Impairment aged between 49 and 89 years (Table 1 and Fig. 2). All participants performed a memory task that indexed 4 components of memory: pattern separation (discriminating two similar items); object recognition (identifying two identical items); novelty detection (identifying new objects) and; spatial rotation sensitivity (identifying two-dimensional rotation about a point of two otherwise identical objects) (Fig. 1b **)**.

**Table 1:** Demographics.

	Frequency	Mean (SD)
Participants	65 (12 MCI)	—
Gender	27 Male	—
Age	—	69.06 (8.57)
IQ Score	—	115.76 (8.36)
Alcohol Intake (average units per week)		6.04 (6.78)
Mild Head Injury (Yes/No)	8/57	—
Epilepsy (Yes/No)	1/64	—
History of Smoking (Smoker/Ex- Smoker)	3/27	—
MoCA (standard deviation) Whole group/healthy controls/MCI		26.66 (2.67)/27.4 (1.93)/23.42 (3.09)
HVLT delayed recall score Whole group/healthy controls/MCI		8.2 (0.35)/9.1 (0.32)/4.9 (0.66)

MoCA = Montreal Cognitive Assessment (Maximum Score 30). HVLT = Hopkins Verbal Learning Task (maximum score 12). As expected patients with MCI performed more poorly than controls on both the MoCA and particularly on the HVLT delayed recall. All of the above participant demographics were obtained via an informal interview. Any individual with a history of severe head injury would have been excluded from the study at prescreening (none were).

## Results

### Distinct profiles of subfield involvement in different aspects of memory

**Figure 3:**
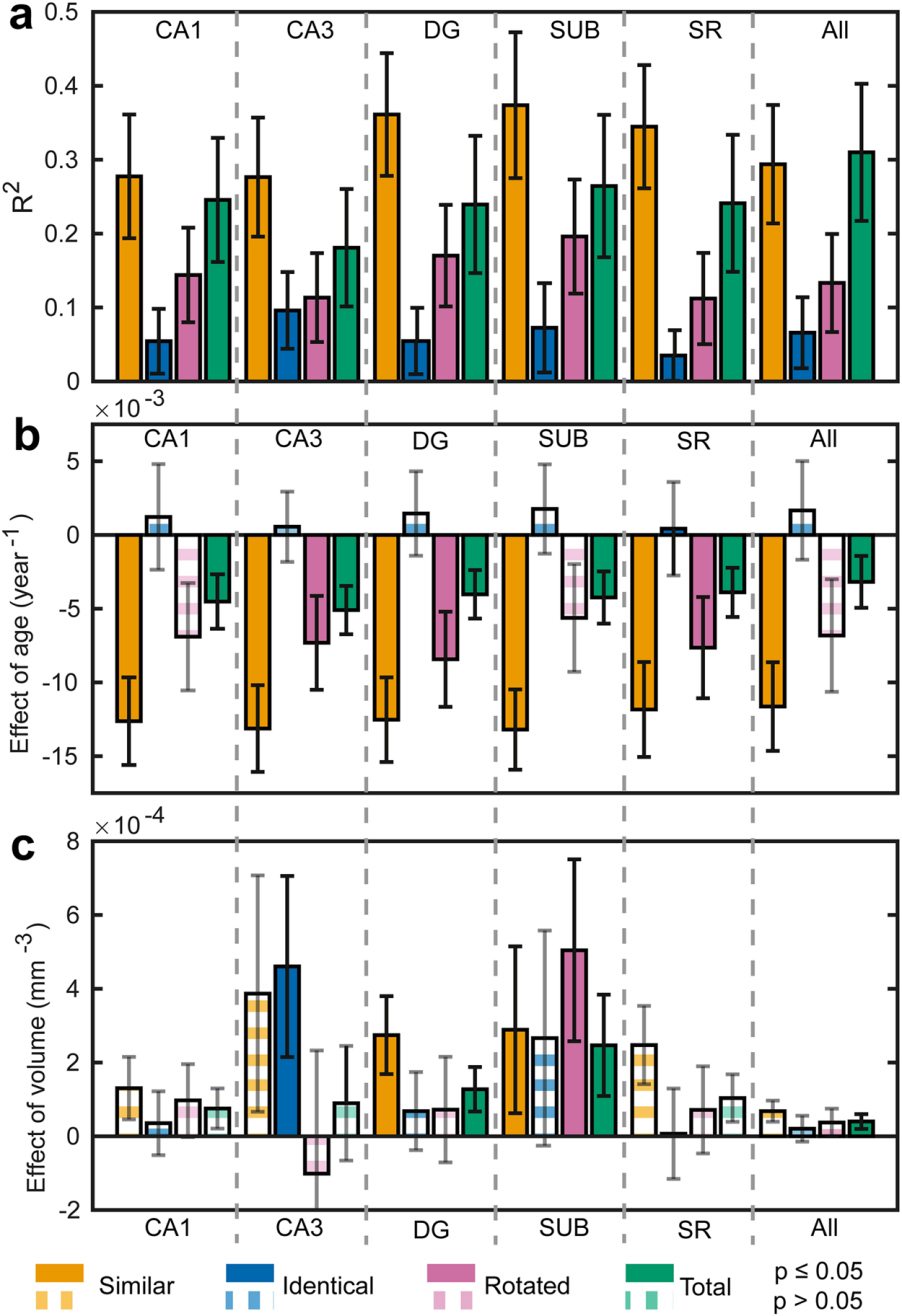
Overview of data across all subfields and tasks analysed. In Fig. 3, we show the correlation coefficients and regression coefficients for the analysis of the effects of all subfield volumes and age on all task scores types. The measured data and fitted models are plotted in detail in Figs 3 and 6. The model P_S,I,R,T_ = Age + V_k_ was fitted for all score types and all subfield volumes separately - k indexes the subfield (or total volume, V = volume). In total, therefore, 24 models were fitted. Panel a shows the R^2^ for each model. Panel b shows the regression coefficient for the effect of age for each model. Panel c shows the regression coefficient for the effect of subfield volume for each model. In all panels, error bars are the 95% confidence intervals derived from bootstrapping the model fit. In panels b and c, bars are shown with solid colour if the p-value is <0.05 and opaque striped if not. Different score types are colour-coded as indicated in the legend beneath panel c. As we were testing specific hypotheses, we did not correct for multiple comparisons. Given we have presented multiple correlations for illustration, we note that, if we were to use Bonferroni correction for multiple comparison, the dentate gyrus relationship with age and task performance remains, but the CA3 results does not. Therefore, there is a very low chance that the relationship between dentate gyrus, age and pattern separation is a false positive, but the CA3 result requires future validation.

**Figure 4:**
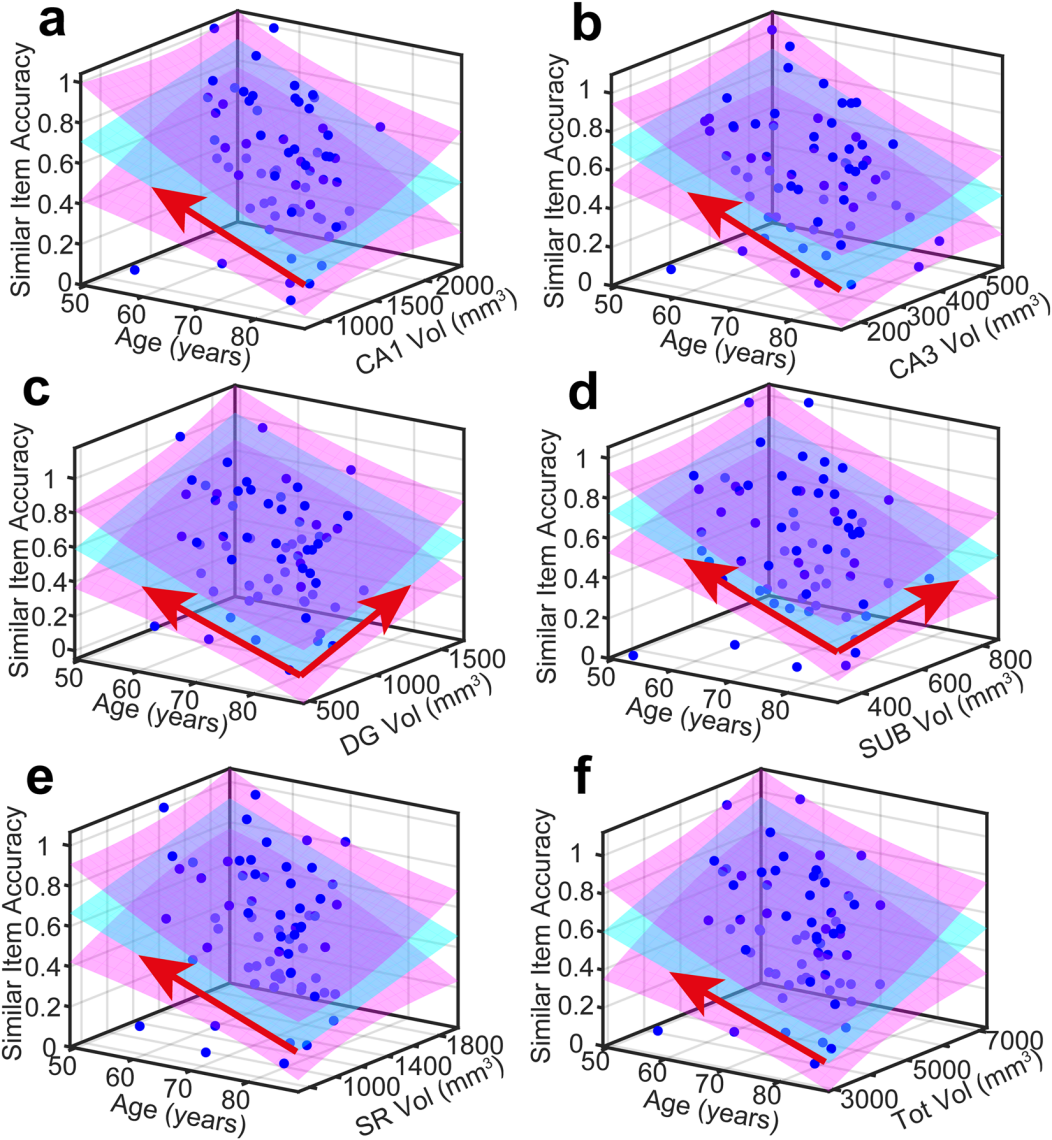
Measured data and quality of model fit for Similar item accuracy (*P*_*S*_). In all panels, measured data is plotted as blue dots, the P_S_ = Age + V_k_ model is cyan and 95% (functional) prediction bounds in magenta. A red arrow projecting over either the Age or Volume axis denotes a significant effect in that dimension at the 0.05 level.

**Figure 5:**
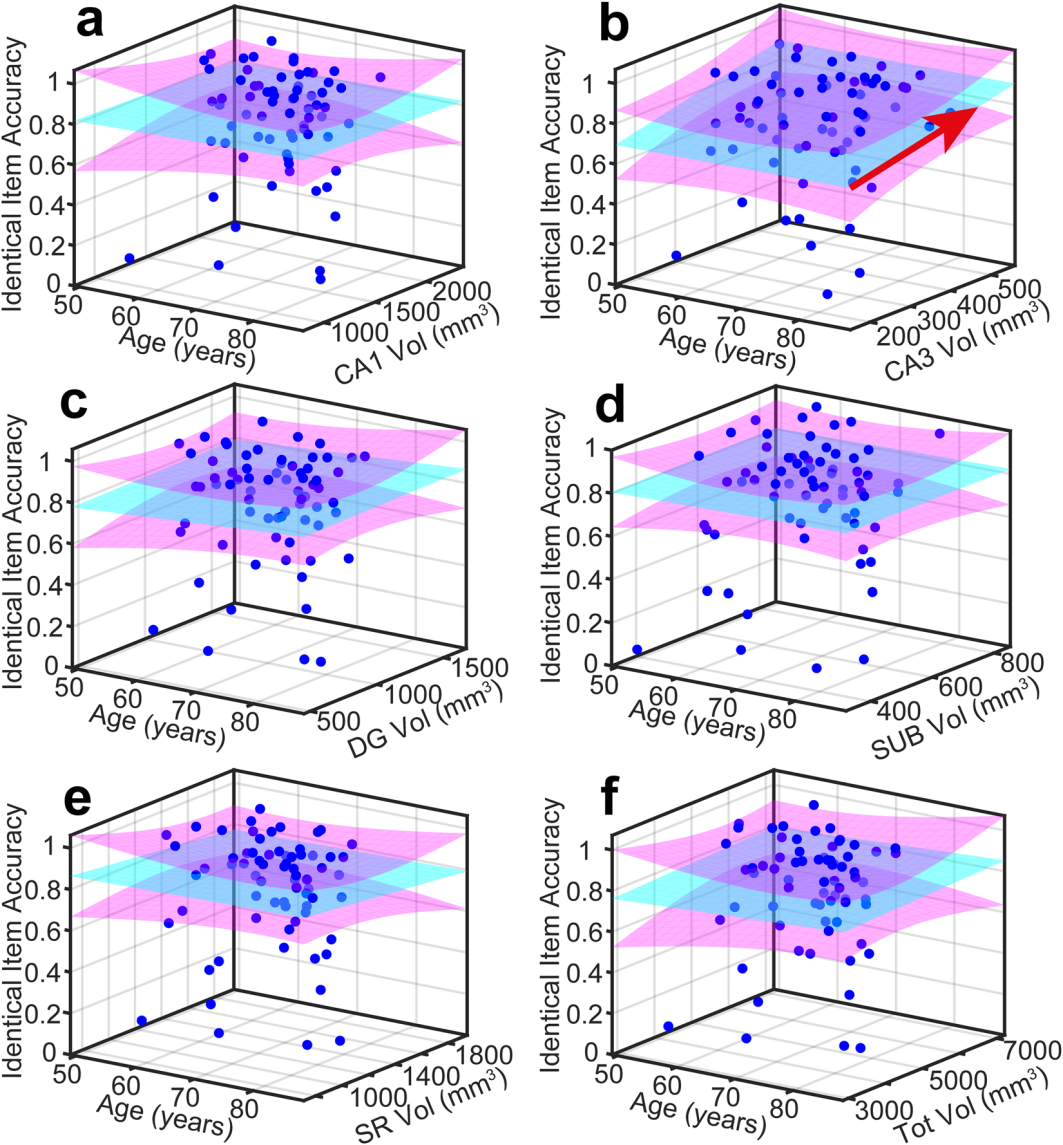
Measured data and quality of model fit for Identical item accuracy (*P*_*I*_). In all panels, measured data is plotted as blue dots, the P_S_ = Age + V_k_ model is cyan and 95% (functional) prediction bounds in magenta. A red arrow projecting over either the Age or Volume axis denotes a significant effect in that dimension at the 0.05 level.

In Figs 3–5, we present our results on investigating the combined effects of age and subfield volume for each task outcome and each subfield volume (raw accuray scores are shown in Figure S1). Our task resolves the hippocampus into functionally distinct domains, the volume of each predicting a different task outcome but with a conserved effect of age on each task outcome. The effect of age is remarkably consistent for each task, but has a distinct effect on each type of task score. This has the important ramification that the distinct task scores “similar”, “identical”, “rotated” are likely to be probing different processes. The effect of age is significant in all subfields and the total hippocampus for similar item accuracy, which we use as our measure of behavioural pattern separation. There is no significant effect of age on identical item accuracy (our measure of object recognition) anywhere. There is a significant effect of age on rotated item accuracy in the CA3, dentate gyrus and stratum radiatum/stratum lacunosum/stratum moleculare, but the effect is smaller than that of similar item accuracy (pattern separation). There is also an effect of age on total score in all subfields, but it is a comparatively weak effect.

Among the different volumes, only dentate gyrus and subiculum show an effect on similar item accuracy at the p < 0.05 level, reflected in the high R^2^ for the model in the dentate gyrus and subiculum subfields compared to the R^2^ for other task scores These data and their fitted model are shown in Fig. 4.

An effect of volume on rotated item accuracy was also detected for dentate gyrus and subiculum and no other subfields. However, the R^2^ was lower than for the similar item model by a factor of about 2. Classification of rotated items (that we would consider reflects coordinate mapping of an object to its location in space, an aspect of pattern separation) also seems also to be localised to dentate gyrus and subiculum.

An effect of volume on identical item accuracy was detected only for CA3, localising pattern completion to this subfield alone. This was a relatively weak effect (despite reaching significance at p < 0.05). Subiculum missed significance only slightly. These data and their fitted model are shown in Fig. 5.

An effect of volume on total score, across the whole behavioural task, was detected for subiculum and total hippocampal volume. Notably, the total hippocampal volume showed an effect only for the total score and not individual components of the task, which is further evidence of functional localisation within the hippocampus.

Therefore, in total, our data localise pattern separation to dentate gyrus and subiculum, object recognition to CA3 and coordinate mapping of an object to dentate gyrus and subiculum. Age has a significant effect on pattern separation but not object recognition. Our intepration of this is the dentate gyrus and CA3 have specific roles in pattern separation and object recognition and subiculum appears to have a generic role that spans these different task requirements.

Having delineated the effect of age and regionalised pattern separation to the DG (with lesser involvement of the SUB), and object recognition uniquely to the CA3, we next sought to investigate whether age or volume has a mediating effect (where the effect of age is significant at all), and further investigate the relative importance of DG and SUB.

### Dentate gyrus fractional volume correlates with pattern separation accuracy and reaction time; it is the site and rate-limiting step of pattern separation

A stepwise regression model for similar item accuracy *P*_*S*_, using all subfield fractional volumes (normalised to TBV) and age as initial inputs, also revealed dentate gyrus as the only significant predictor of pattern separation accuracy (*F*[1,63] = 7.512, *p* = 0.008, dentate gyrus volume *Beta* = 0.326, *p* = 0.008, *R* ^2^ = 0.107). This provides further evidence for functional localisation of pattern separation to the dentate gyrus.

We also considered whether processing in the dentate gyrus was a rate limiting step for pattern separation performance. Response time to correctly identified similar lures was also predicted by dentate gyrus volume and not by other subfield volumes (*F* = 13.617, *p* = 0.000, *R* ^2^ = 0.190: dentate gyrus volume *Beta* = −0.436, *p* = 0.000). This supports our proposal that integrity of dentate gyrus is crucial for both accuracy and response speed of pattern separation.

### Hippocampal white matter doesn’t enter into a model in which dentate gyrus is present

In order to establish whether dentate gyrus integrity, rather than connections from neocortex to hippocampus, is truly the rate limiting factor in pattern separation, we segmented the hippocampal region stratum radiatum/stratum lacunosum/stratum moleculare, a brain region that predominantly comprises axons and synapses of the perforant pathway (the main pathway from neocortex to hippocampus), adjacent to dentate gyrus. Stepwise regression demonstrated that dentate gyrus volume significantly predicted both accuracy and reaction time, but the adjacent predominantly white matter region did not (accuracy: *F* = 7.512, *p* = 0.008, *R* ^2^ = 0.107: SR/SL/SM volume *Beta* = −0.076, *p* = 0.688, reaction time *F* = 13.617, *p* = 0.000, *R* ^2^ = 0.190: SR/SL/SM volume *Beta* = −0.098, *p* = 0.608) – Fig. 3e. Thus we propose that activity within the dentate gyrus itself, rather than its white matter connections, defines pattern separation ability and that the normalised subfield volume is a proxy measure of the proportion of available neurons working efficiently within the dentate.

### DG fractional volume, not subiculum volume, is the mediator of pattern separation accuracy

**Figure 6:**
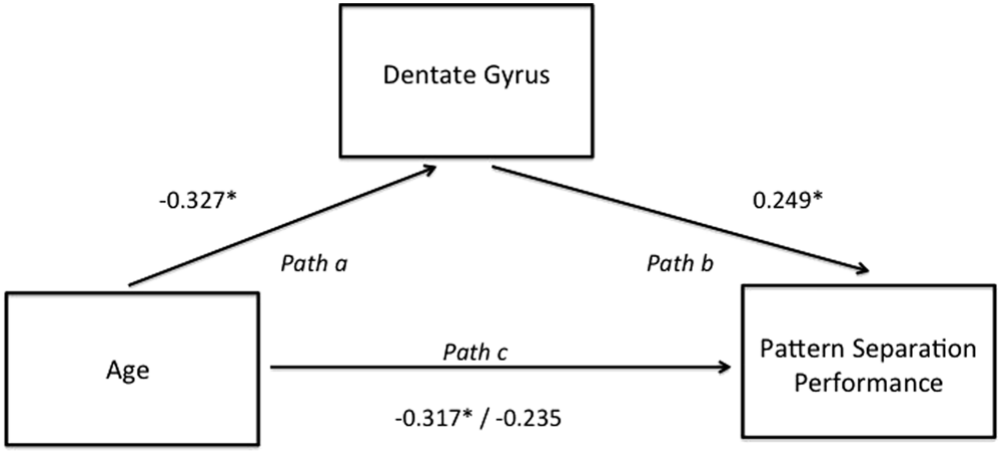
Mediation analysis for age, dentate gyrus and pattern separation performance. Dentate gyrus mediates the effect of age on pattern separation accuracy. Numbers represent standardised beta coefficients and the value after the forward slash indicates the standardised coefficient after the inclusion of the mediator (dentate gyrus). *P < 0.05.

Since both age and dentate gyrus volume are correlates of pattern separation (Figs 3b,c and 4c), to further investigate how age affected pattern separation through its effects on dentate gyrus, mediation analysis was performed. In sequential linear regressions in which the effect of volume was modelled in the first step and age independently in a second step (Fig. 6, *path b*), the dentate gyrus fractional volume was shown to significantly predict pattern separation performance (*Beta* = 0.249, *p* = 0.048, Regression: *F*[2,62] = 5.731, *p* = 0.005, *R* ^2^ = 0.156), whereas, age no longer had a significant association with pattern separation performance (*Beta* = −0.235, *p* = 0.061). Therefore, a significant proportion of the effects of age on pattern separation performance did indeed depend on dentate gyrus volume. Note that there was no mediation of the effects of age by subiculum volume to explain pattern separation performance – when the same analysis was performed for subiculum, age was the only predictor of pattern separation (Regression: *F*[2,62] = 4,276, *p* = 0.018, *R* ^2^ = 0.121, Age: *Beta* = −0.290, *p* = 0.020, Subiculum: *Beta* = 0.003, *p* = 0.233). Therefore, we suggest the effects of age on pattern separation memory are mediated through dentate gyrus atrophy.

Importantly also, as shown in Figs 3 and 4a,f, CA1 and total hippocampal volume were strongly negatively correlated with age, but did not predict pattern separation performance (*Beta* = 0.075, *p* = 0.643) arguing against failure in pattern separation being a general effect of ageing on multiple hippocampal regions (Figs 3a and 4a). Rather we have shown that specific age-related impairment of dentate gyrus mediates a decline in separation performance.

### CA3 fractional volume correlates with accuracy of object recognition

As a further test of which subfield volume was the best predictor of object recognition (not requiring pattern separation), we used stepwise regression, in which terms were systematically eliminated if they failed to make an adequate contribution to predicting object recognition (identical item accuracy), and interaction terms added if needed. The starting model contained all subfield volumes. Age was not modelled as it was shown not to have a significant effect (Figs 3b and 5a–f). Stepwise regression was significant (*F*[1,63] = 5.108, *p* = 0.027) and CA3 was the only significant predictor of object recognition performance (*Beta* = 0.274, *p* = 0.027, *R* ^2^ = 0.075) (consistent with Fig. 5b). Dentate gyrus did not trend towards predicting performance in the object recognition component of the task (*Beta* = 0.104, *p* = 0.411) (consistent with Fig. 5c). This provides further evidence that object recognition is functionally localised to CA3.

Thus we have demonstrated that the dentate gyrus, distinct from CA3, is critical for pattern separation and CA3 underpins object recognition when pattern separation is not required. It is important to note that these significant relationships exist when we analyse both the healthy control and MCI participants together, but are not present when either group is analysed separately (Figure S2).

## Discussion

We have provided evidence that in the human brain, pattern separation is functionally localised to the DG hippocampal subfield whilst object recognition, not requiring pattern separation, is functionally localised to the CA3 hippocampal subfield. Such selective relationships between behaviour and individual subfield volume within the human hippocampus confirm hypotheses generated from the animal literature and the validity of inference about human functional neuroanatomy from animal models of memory. Pattern separation was particularly susceptible to the effects of increasing age and volume reduction in the dentate gyrus critically determines the effects of ageing on pattern separation. Importantly, no other hippocampal subfield was as strongly associated with pattern separation performance and neither dentate gyrus volume nor age predicted performance in the object recognition task, where pattern separation was not required. Even white matter normalised volume adjacent to the dentate gyrus did not define accuracy and reaction time to similar items as well as dentate gyrus itself. On the basis of these findings, we support the animal models where pattern separation depends on the dentate gyrus separating overlapping inputs and feeding forward to the CA3 where pattern completion and object recognition occur leading to memory recall through interaction with higher cortical areas (Fig. 1b)^4^.

We included both healthy older people and those with MCI in our analyses to ensure a range of cognitive ability. The rationale behind including both healthy older people and those with MCI was to increase the range of performance such that we could test our hypotheses about structure function correlations. Most people with MCI do not progress to Alzheimer’s disease even after 10 years^24^ – although initial conversion rates are 10–15%, this dwindles over time such that a significant proportion of people with MCI never develop Alzheimer’s disease. Sixty-two per cent of healthy older control participants in the Alzheimer’s Disease Neuroimaging Initiative have positive amyloid in CSF suggestive of Alzheimer’s disease pathology^25^. Therefore, we view MCI and healthy ageing as representing a spectrum of cognitive abilities impacted by both ageing and Alzheimer’s disease (and perhaps other factors). We do not yet know which participants in this dataset (with or without MCI) will progress to dementia. It is important to note that the groups are not compared directly; rather the relationships between behavioural performance, age and hippocampal subfield volumes are interrogated across all participants, both MCI and healthy older people. Results largely overlapped (see Figure S2 for a scatter plots differentiating MCI and healthy participant data) between the two groups and boot strapping used in our first analysis helps to ensure that outliers are not driving our significant relationships.

We used two complementary analysis techniques to pinpoint the role of hippocampal subfields. First we took an overview of subfield and whole hippocampal volumes, age and pattern separation, rotation and object recognition as well as total performance on our behavioural task of memory (Figs 3–5). This had the advantage of demonstrating that individual subfields explained performance better than total hippocampal volume and demonstrated distinct patterns of subfield involvement individual tasks in line with our hypothesis. We also allowed objective criteria to determine the most probable contributors to the various task outcomes by stepwise regression. We found that the two analysis techniques converged to demonstrate distinct subfield associations with cognitive tasks.

Neurophysiology from animal models points to a trisynaptic pathway from entorhinal cortex to dentate gyrus then to CA3 and on to CA1 underpinning memory, with each area within this network contributing a distinct set of properties^26^. This adds a level of complexity when trying to establish critical functions of distinct subregions within the network subserving pattern separation and object recognition – each function is dependent not only on the area performing the critical computation, but also the connecting structures to and from that area. Here we used the principle that the integrity of the area *critical* for the computation underpinning pattern separation will define behavioural performance more precisely than integrity of other connecting areas. This is particularly pertinent when one considers that dentate gyrus better predicted pattern separation performance that the adjacent white matter containing the performant pathway. This result appears at odds with the finding that the perforant pathway integrity, assessed using diffusion tensor imaging, is very sensitive to the effects of age and age-related cognitive decline^27^. However, we are not arguing that the perforant path has no relationship to age-related cognitive decline, just that the dentate gyrus is the critical area performing the pattern separation computation. It will be interesting in future to differentiate between perforant pathway and dentate gyrus function perhaps through combined use of volumetrics and diffusion tensor imaging.

One unexpected finding from our data was a significant relationship between subiculum volume and pattern separation, rotation and total performance in the task. Subiculum is known to contain cells sensitive to direction of movement and head direction, cells that undergo synaptic plasticity, and the anteromedial subiculum in humans is through to have a role in scene discrimination (independent of viewpoint)^28,29^. Interestingly subiculum was the only subfield volume to predict the ability to perform the rotation detection task, perhaps in keeping with a role in coordinate mapping and spatial memory, but we also note broader associations than other subfields within our task. Given the potential broad-range functionality of subiculum, we propose that, at least in terms of our task, subiculum has a more generic role in memory rather than specifically object recognition or pattern separation. We consider the result demonstrated here as hypothesis generating for future work on the function of the subiculum in human memory.

The focal effects of age on the dentate gyrus function have exciting implications for the potential to enhance cognition in older adults. In recent years, the impact of impaired memory on quality of life has been recognised. Emerging evidence suggests interventions, such as exercise^30^ and dietary flavanols^31^, putatively enhance dentate gyrus-dependent function to improve memory. In addition, hippocampal subfield volumes could be used to help stratify treatment for patients with memory impairment towards pharmacotherapies that might slow progression of Alzheimer’s pathology or ameliorate impact of ageing on cognition. Enriched understanding of the role of the dentate gyrus and CA3 in human memory will improve design of studies to target and monitor the effects of interventional trials for memory disorders. More widely, anxiety disorders may also be associated with overgeneralisation due to failure of pattern separation^32^ and hippocampal subfields may have differential contributions to emotional processing^33^. Fractionating hippocampal subfield function through detailed structural imaging and focussed behavioural paradigms is an emerging technique for better understanding of human memory^34,35^.

### Limitations

We have limited our analysis to the hippocampus because of the wealth of animal data supporting a role for the hippocampus in human memory. However, there may be regions outside the hippocampus, such as the white matter connections in the limbic tracts^36^ that contribute to our understanding of human memory, but are outside the scope of this study. One could further question the relevance of structure-function correlations that are just below the cut off for statistical significance (e.g. the similar performance bar in Fig. 3 for CA3). We have assumed that non-critical nodes in the trisynaptic loop will potentially influence performance as they provide a conduit for signal to reach the critical node, but that their volume/function statistical relationship will be lower compared with a critical node. Our data do not discriminate this assumption from, for example, a functionally contributory, but less important role for CA3 in pattern separation than dentate gyrus. This is an area for future exploration. Further, our analyses assume that the task is being carried out by participants in a predictable way, consistent across individuals and time, whereas, there will be differing amounts of noise between individuals related to their response to perceived errors, motivation and attention that are not captured in our analysis. We note that there are many available hippocampal segmentation protocols from either T1 or T2 MR images and the field is moving towards validating an optimised protocol. Our protocol was developed using T2 images generated from a Carr-Purcell-Meiboom Gill multi-echo image set as input in line with a prominent protocol available at the start of our project^37^ with Duvernoy hippocampus atlas as reference for neuro-anatomy. We wish to stress that the segmentation by Wood *et al*. is optimised for CPMG T2-images as input for a wide age range, and consequent contrast changes^38^, typical in the participant cohort in this study. We hope that by sharing the precise details of our technique, we can feed into the efforts to harmonise protocols.

Broadly, although there are limitations and assumptions in our study, the effect of these would be to minimise the chance of us showing significant structure function correlations and are very unlikely to have generated false positive findings. However, one potential confound we cannot exclude on the basis of our data is that Alzheimer’s disease pathology, more prominent in the MCI group, is driving the correlations. We think this is unlikely as boot strapping minimises the likelihood that outliers are driving the results and CA3/dentate gyrus are not the first subfields to be affected by Alzheimer’s pathology (neuropathological study suggests CA1)^39^. However, data do not generate significant relationships if the groups are analysed separately and, therefore, future larger groups with clearly defined biomarker profiles are needed to exclude this possible confound.

## Conclusions

Dentate gyrus and CA3 subfields of the hippocampus have distinct roles in human memory and hippocampal subfields segmentation is a valuable technique for fractionating structure function correlations in small areas of the human brain.

## Materials and Methods

### Participants

Participants were recruited from the following local and national volunteer registers: Join Dementia Research, BRACE charity, Avon and Wiltshire Mental Health Partnership Trust, ReMemBr group research database and North Bristol NHS Trust. 70 participants gave informed consent to take part in the experiment. Of these, 2 people were lost to follow up and we did not have complete pattern separation data and 3 people were not able to have MRI scans due to previous implants. Of the sample of 65 with complete testing, 53 were healthy aged individuals who had no self-reported memory impairment and 12 people had a recent diagnosis of MCI (Table 1). MCI was diagnosed at our own and other local memory services using standard criteria^40^ and, for inclusion, all MCI had to have had memory complaints and/or score more than 1 SD below expected for age on a test of memory. Note that this includes both amnesic only and multidomain MCI and we did not differentiate between these participants in this analysis. We planned to exclude those with other significant neurological illnesses likely to interfere with test performance (but in fact no-one had to be excluded on this basis). All participants underwent a screening interview to assess demographic factors thought to impact on cognitive decline (IQ, history of head trauma, epilepsy, smoking and alcohol intake) e.g. ref.^41^). All participants retained the capacity to consent to research and ethics approval was granted by the Research Ethics Committee of North Bristol NHS Trust. All experiments were carried out in accordance with ethical and NHS governance guidelines and approvals. University of Bristol agree in principle to anonymised data sharing with external researchers and each data request is assessed individually.

### Experimental Design

Participants completed cognitive testing and an MRI scan over 2 visits, within an 8 week time frame. This cross-sectional study was a planned component of our larger hippocampal imaging project where longitudinal assessments are ongoing, focussing on the sensitivity to cognitive decline of hippocampal subfield imaging and other MRI metrics, combined with cognitive tests of long term memory.

### Measuring Pattern Separation

We utilised a task of visual pattern separation, an adaptation of the Mnemonic Similarity Task (MST), well described in the literature^42–44^. During the task, colour photographs of everyday objects were displayed on a computer screen one at a time for 2500ms. The task consisted of 92 novel items, 16 items identical to a previously presented novel item, 16 items that were similar to a previously presented novel item (lures) and 16 items identical to a previously presented novel item but rotated either clockwise or counter-clockwise by 40 degrees. Items were presented in pseudo-randomised order with “identical”, “similar” and “rotated” items coming within 4 to 40 items of their novel counterparts. Participants were asked to respond to each item presentation with a button press corresponding to one of the four categories; “new”, “identical”, “similar” or “rotated”, they were given unlimited time to respond. The “rotated” category was added to the previously used versions of this task as many of the similar items differed in terms of their spatial rotation (as well as other factors). We used the rotated category to help define whether subfield associations were related to the ability to distinguish object orientation rather than changes in object identity. All 140 items were presented in one block lasting approximately 20 minutes. A brief practice task helped participants orient themselves to the demands of the task. In particular we were interested in participant’s ability to correctly encode the similar items as distinct representations from their novel counterparts and subsequently categorise the lures as “similar” and the ability to detect identical objects. This ability to discriminate visually similar vs identical objects is thought to represent pattern separation and object recognition (although these are assumptions as one cannot know the computational basis of behaviour in each individual) in the hippocampus, the focus of our hypothesis.

The output signal of the task comprised the set of classifications made by the participant and the times taken to do so. From this set of responses, we defined the quantity “pattern separation performance”, *Ps*, as the total number of correct classifications in response to similar lures. We defined the quantity “object recognition performance”, *P*_*OR*_, as the total number of correct classifications in response to identical lures. Corresponding mean reaction times for all similar and identical lures were also calculated.

Throughout the paper, data are presented for similar items as a measure of pattern separation, identical items as a measure of object recognition and rotated items as a measure of detection of coordinate remapping. However data in the new item category were too clustered around perfect performance to obtain meaningful results, not normally distributed even after transformation using Shapiro Wilk statistic, and are therefore not analysed. Note that we did not attempt to correct for response bias (the tendency to choose one response button over the others for reasons that are not task related) because: i) the new items were presented far more often thus potentially generating a response bias and ii) we were expecting some variation in response with age (we are expecting change in bias from similar to identical) – pressing the identical button to a similar stimulus with increasing age is an experimental outcome that would be diluted by correcting for response bias if one considers that all the remaining categories – identical, rotated, similar would be possibly be affected by an age related bias).

### Imaging Protocol

MR images were acquired using a 3 T Siemens Magnetom Skyra scanner with a 32-channel head coil. Volumetric T1-wrighted images were acquired using a 3D MPRAGE sequence (TR = 2200ms, TI = 900ms, TE = 2.42ms, alpha flip angle = 9°, FOV = 220 × 184 × 230mm, resolution = 0.34 × 0.34 × 1.6 mm^3^ following 2-fold interpolation) with an acquisition time of 5.25 minutes. T2 images were acquired using a 2D multi-echo spin echo sequence in the oblique plane in which the long axis of the hippocampus was perpendicular to the coronal plane (TR = 5500 ms, TE = 12ms, echo spacing = 12 ms, 10 echoes, FOV = 184 × 218 × 58 mm^3^, in-plane resolution = 0.34 × 0.34 × 1.72 mm^3^ after 2-fold interpolation in-plane and inclusive of 15% slice gap). Acquisition time was ~12 minutes. The magnitude images corresponding to an entire echo train were then summed.

### Image Post-processing

In order to calculate Total Brain Volume, FSL’s BET (brain extraction tool)^45^ was applied to T1-weighted images to remove the skull and non-brain tissue. The resulting images were visually inspected and altered manually as required. The resulting volume of the resulting images was calculated using fslstats to give TBV in mm^3^. All processing steps were completed using FSL software (https://fsl.fmrib.ox.ac.uk/fsldownloads/).

### Recognition of Hippocampal subfield boundaries

Using an advanced MR protocol we previously described^23^, the T2-weighted images were used to label five separate hippocampal subfields along the entirety of the anterior-posterior axis: the cornu ammonis (CA) 1, CA2, CA3, dentate gyrus (DG) and subiculum, as well as the combined stratum radiatum/stratum lacunosum/stratum moleculare (SR/SL/SM). T2-weighted images were used for this purpose due to the superior contrast displayed by this type of image making internal structural boundaries easier to recognise^37^. In brief, this protocol uses GM and WM contrast differences to recognise external hippocampal boundaries and the internal SR/SL/SM subfield. To delineate the subiculum and the DG the protocol uses both image contrast and anatomical markers. To label the boundaries of the CA subfields (CA1, CA2 and CA3) it uses geometric rules governed from post mortem data and protocols written from ultra-high field data^34,37,46^. Subfields were all labelled manually on both the left and right hemispheres using FSL software and their absolute volumes calculated using FSL. Global hippocampal volumes were calculated by summing the volumes of the six subfields. Typical segmentation is shown in Fig. 2.

### Testing Segmentation Protocol Reliability

To test the reliability of the segmentation protocol a second rater, trained in recognising the subfield boundaries described in the protocol, re-segmented four hippocampi. Rater reliability was compared using intraclass correlation coefficient (ICC), performed on SPSS. The ICC results, obtained using a 2-way mixed model assessing absolute agreement, for inter-rater reliability indicated high reliability for both total hippocampal and hippocampal subfield volume measurements. The average measure ICC was found to be 0.993 with a 95% confidence interval from 0.986 to 0.996 (F(62,62) = 168.91, p < 0.0001). This reliability is at least as good as other published segmentation protocols.

### Data analysis

We used two approaches to analysis – first we computed an overview of all subfields and task dimensions of interest and next we examined which quantities had most explanatory power to explain pattern separation and object recognition.

For the first analysis, to investigate the effects of age and subfield volumes upon the different categories of task score, we fitted for each type of task score and each subfield (and total hippocampal) volume the regression model

1 $${P}_{S,I,R,T}={\rm{\beta }}0+{{\rm{\beta }}}_{1}Age+{{\rm{\beta }}}_{2}{{\rm{V}}}_{{\rm{k}}}$$

where S = similar, I = identical, R = rotated, T = total score and *k* indexes the particular volume whose effect is modelled (*k* = CA1, CA3, DG, SUB, SR, Total volume). β_1,2,3_ are the regression coefficients, *V*_*k*_ a hippocampal subfield (or total) volume. Therefore 24 models were fitted, each with 3 coefficients.

95% confidence intervals were derived from bootstrapping the model fit with 1000 iterations to observe the pattern performance across different aspects of memory, subfields as well as total scores and whole hippocampal volumes.

Stepwise regression analyses were conducted using IBM SPSS Statistics software, version 23. Where data were not normally distributed (tested using Shapiro Wilk statistic in SPSS), we performed transformation (conversion to z score) to mitigate skew and were able to use converted scores for parametric statistics (except for the performance in the new item identification which remained too highly skewed to be meaningfully analysed). Therefore our analyses include parametric statistic on behavioural performance (converted to z scores for identical, similar and rotated categories of behavioural task), brain volumes, and demographics. Note that the SL/SR/SM areas was not included in the initial subfield analysis as it incorporates more than one subfield and is predominantly white matter.

For the second analysis, to determine the quantities with the greatest explanatory power of the task outcomes, we used stepwise regression. The set of models took the initial form

2 $${P}_{S,I,R,T}={{\rm{\beta }}}_{0}+\sum _{{\rm{k}}}{{\rm{\beta }}}_{{\rm{k}}}{{\rm{V}}}_{{\rm{k}}}$$

with terms having the same meaning as before. Initially, the model contained the age plus fractional volumes (normalised to TBV to control for differences in head size) of CA1, CA2, CA3, DG and SUB.

The comparison between dentate gyrus and white matter of SR/SL/SM was made using the model.

3 $${P}_{S}={{\rm{V}}}_{{\rm{DG}}}+{{\rm{V}}}_{\text{SR}/\text{SL}/\text{SM}}$$

P_S_ = Pattern Separation, V = nomalised volume, DG = Dentate gyrus, SR/SR/SM = stratum radiatum/stratum lacunosum/stratum moleculare.

The mediation analysis was performed using sequential linear regressions^47^.

## Electronic supplementary material

Supplementary information
