# Supplementary material for: The impact of ageing reveals distinct roles for human dentate gyrus and CA3 in pattern separation and object recognition memory
Source: Sci Rep. 2017 Oct 25;7:14069. doi: 10.1038/s41598-017-13853-8 (PMC5656671; doi:10.1038/s41598-017-13853-8)
Supplement: Supplementary file 1 — Supplementary information [file 41598_2017_13853_MOESM1_ESM.doc]

The impact of ageing reveals distinct roles for human dentate gyrus and CA3 in pattern separation and object recognition memory.

Serena E. Dillon* ^1,3^, Demitra Tsivos* ^1,2^, Michael Knight ^3^, Bryony McCann ^3^, Catherine Pennington ^1, 2^, Anna I. Shiel ^4^, Myra E. Conway ^4^, Margaret A. Newson ^1,3^, Risto A. Kauppinen ^3^ and Elizabeth J. Coulthard ^1, 2^

Affiliations

1 Bristol Institute of Clinical Neuroscience, North Bristol NHS Trust, Southmead Hospital BS10 5NB

2 School of Clinical Sciences, University of Bristol, Learning and Research Building, Southmead BS10 5NB

3 School of Experimental Psychology, University of Bristol, 12A Priory Road, BS8 1TU.

4 Department of Applied Science, University of West of England, Frenchay Campus, Coldharbour Lane, Bristol BS16 1QY

*Joint first authors

Corresponding author: Dr Elizabeth Coulthard, School of Clinical Sciences, University of Bristol, Learning and Research Building, Southmead BS10 5NB. [elizabeth.coulthard@bristol.ac.uk](mailto:elizabeth.coulthard@bristol.ac.uk) 0117 4148238

Keywords: hippocampus, memory, dementia, MRI, pattern separation

**Table S1 Table of Average Sizes of Subfield Volumes (mm3)**

| **Subfield** | **Mean Normalised Volume** | | **Mean Raw Volume (mm3)** |
| --- | --- | --- | --- |
| **CA1** | 623.83 | 839.05 | |
| **CA2** | 31.15 | 41.76 | |
| **CA3** | 137.32 | 185.39 | |
| **DG** | 404.21 | 544.28 | |
| **SUB** | 210.30 | 281.84 | |
| **Total** | 1406.82 | 1892.32 | |

**Table S1 Subfield Sizes.**
Normalised volumes = raw volume/Total Brain volume*1000.

**Figure S1 Overall accuracy rates for different task conditions in healthy controls and patients with mild cognitive impairment**


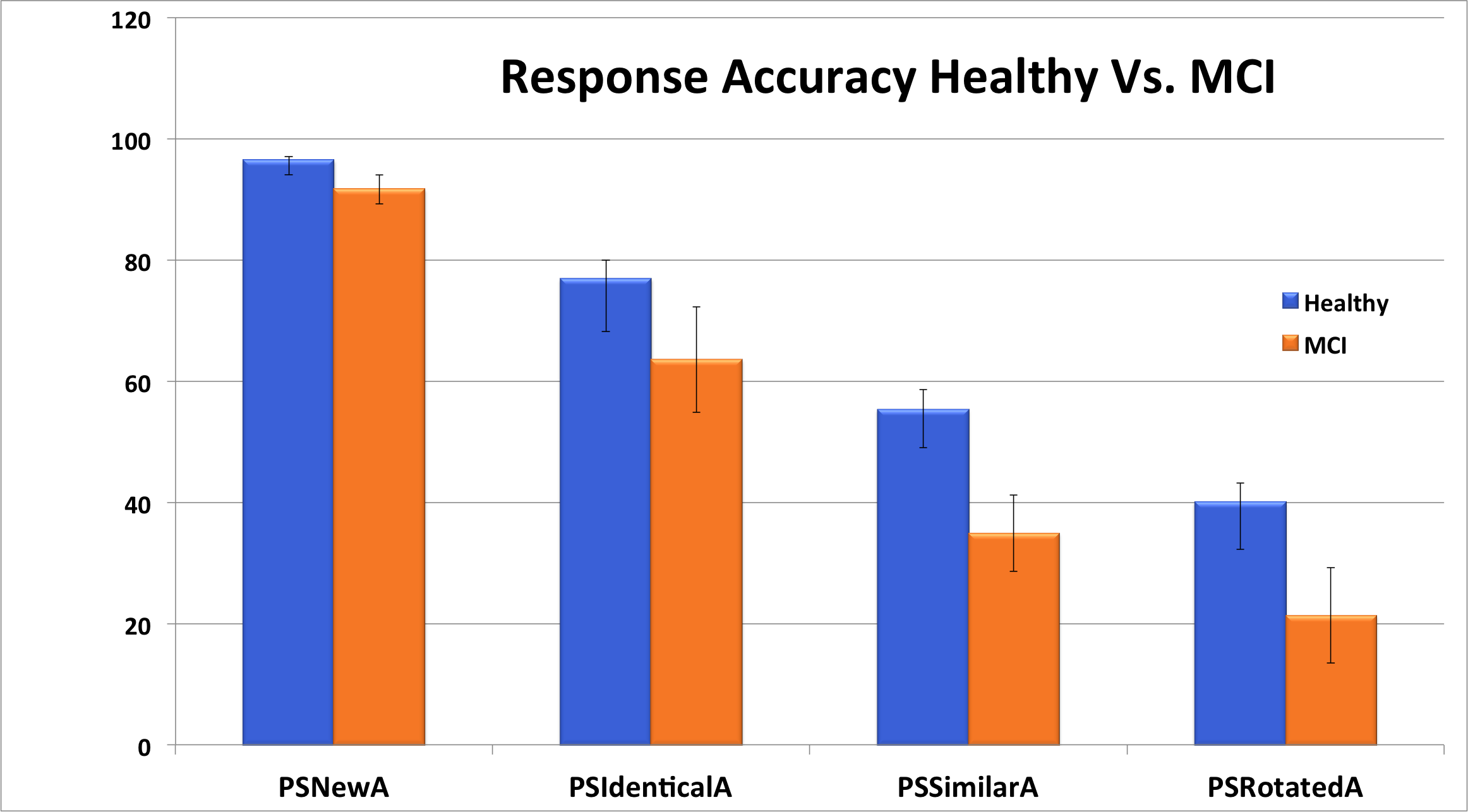
**Legend** Graph shows percentage accuracy and standard error bars. Accuracy differed between different task conditions (accuracy from highest to lowest: New>Identical>Similar>Rotated). Accuracy of identifying new items clustered around ceiling performance (90-100%) – data were not parametrically distributed even after trasnformation and so further analyses were not performed on these data. Given these performance differences, we were alert to the possibility that task difficulty might explain differences in structure function correlations (i.e. it is possible that a brain area is more involved in more difficult tasks regardless of other task demands). It is not possible to completely exclude this as a confound, but if this were the case, we would expect that any such brain area would be progressively more related to performance as ask difficulty increased. **Figure 3** does not show any such relationship. We also note that performance in the rotated category was around chance in the MCI group and only just above chance in the healthy group. This limited the inference we have made about the rotated performance, particularly the comparisons with similar performance which was clearly above chance.

**Figure S2 Scatter plots demonstrating the relationships of dentate gyrus, CA1 and CA3 volumes with age (grey), pattern separation accuracy/response time (black) and object recognition (blue) in MCI (open circles) and healthy participants (filled circles).**


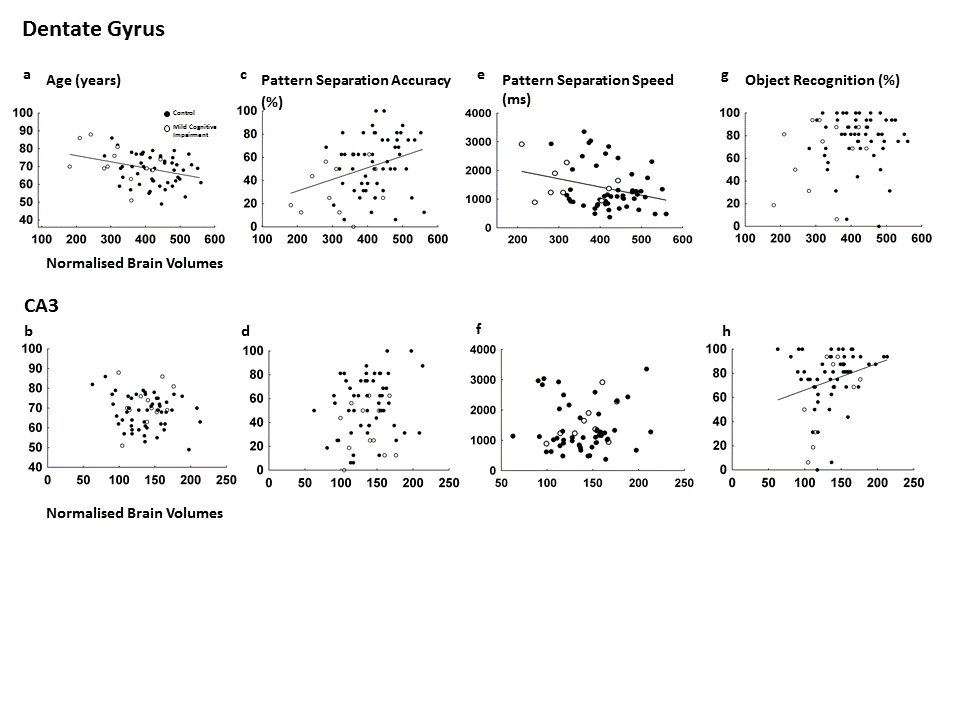

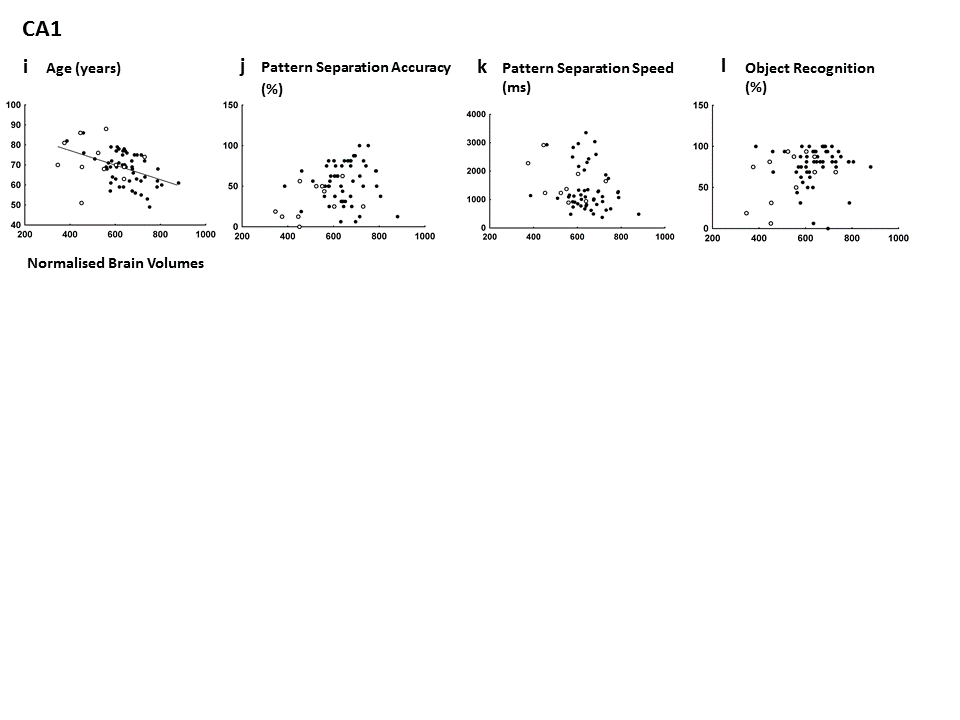


Note that data for MCI and healthy groups overlap with MCI tending to have poorer performance. Statistical tests run in either group alone yielded only trends in the directions of the whole group analysis.

**Legend for Figure S2**

Visual inspection shows that MCI and healthy control participant data overlaps. Dentate gyrus volume declines with age (a) whereas CA3 does not significantly (b). Dentate gyrus volume predicts pattern separation accuracy (c) and reaction time (e) whereas CA3 does not (d and f). CA3 volume predicts object recognition memory (h) whereas dentate gyrus does not (g). Similar scatter plots are presented for CA1(i-l) showing that CA1 volume declines with age but does not have significant relationships with pattern separation or object recognition. Lines of best fit show where the relationship is significant. All volumes for this analysis are expressed as normalised volumes = ((subfield volume in mm^3^)/(Total Brain Volume in mm^3^)) x 1000.

**ApoE4 analysis**

Given that none of our demographic factors (including age) predicted CA3 volume, we sought to assess whether ApoE status, the most common risk factor for Alzheimer’s disease, would predict subfield volume. We were able to take blood to assess ApoE genotype on a subset of our participant group (n=43). We divided participants into two groups ApoE4 positive (n=18) and ApoE4 negative (n=25).

Amplification of ApoE from the whole blood sample and *Hha*I restriction digest genotyping.

ApoE genotyping was performed using a PCR method, previously described by ^1^. DNA was extracted from 300 µL of whole blood using Ultra Clean Blood Non-Spin Kit (Cambio). DNA was then amplified by PCR using oligonucleotide primers (Eurofins Genomics; upstream primer: 5ʹ- TCC AAG GAG CTG CAG GCG GCG CA -3ʹ, downstream primer: 5ʹ- ACA GAA TTC GCC CCG GCC TGG TAC ACT GCC A - 3ʹ). ApoE was amplified using a PTC-200 Peltier Thermal Cycler at 94 °C for 12 minutes followed by 48 cycles at 94 °C for 1 minute, 65 °C for 1 minute and a final extension of 75 °C for 1 minute. The PCR product was subsequently mixed with 1 µl *Hha*I restriction endonuclease (New England Biolabs) at 37 °C overnight. After digestion each reaction was separated on a 3.5 % Metaphor gel (Lonza) supplied with ethidium bromide. Migration pattern of DNA fragments, visualised by UV illumination, were used to identify a genotype of each sample. ApoE e2/2 is defined by the presence of two fragments, 91 bp and 81 bp, ApoE e4/4 has a unique 72 bp fragment, while ApoE e3/3 lacks the 81 bp and 72 bp fragments. For heterozygotes, there are combinations of different fragments visible on gel ^1^.

Linear regression showed no relationship between subfield volume or behavioural performance and ApoE status.

**Supplementary references**

1 Hixson, J. E. & Vernier, D. T. Restriction isotyping of human apolipoprotein E by gene amplification and cleavage with HhaI*. Journal of lipid resear*c**h** 31, 545-548 (1990).
